# Somatosensory Modulation of Salivary Gene Expression and Oral Feeding in Preterm Infants: Randomized Controlled Trial

**DOI:** 10.2196/resprot.7712

**Published:** 2017-06-14

**Authors:** Steven Michael Barlow, Jill Lamanna Maron, Gil Alterovitz, Dongli Song, Bernard Joseph Wilson, Priya Jegatheesan, Balaji Govindaswami, Jaehoon Lee, Austin Oder Rosner

**Affiliations:** ^1^ Center for Brain, Biology, and Behavior Department of Special Education and Communication Disorders, Biological Systems Engineering University of Nebraska Lincoln, NE United States; ^2^ Tufts Medical Center Division of Neonatology, Department of Pediatrics Boston, MA United States; ^3^ Center for Biomedical Informatics Harvard Medical School Boston, MA United States; ^4^ Division of Neonatology Department of Pediatrics Santa Clara Valley Medical Center San Jose, CA United States; ^5^ CHI Health St. Elizabeth Division of Neonatal-Perinatal Medicine Lincoln, NE United States; ^6^ IMMAP Department of Educational Psychology and Leadership Texas Tech University Lubbock, TX United States

**Keywords:** gene expression, preterm infants, brain, mouth, mechanoreceptors

## Abstract

**Background:**

Despite numerous medical advances in the care of at-risk preterm neonates, oral feeding still represents one of the first and most advanced neurological challenges facing this delicate population. Objective, quantitative, and noninvasive assessment tools, as well as neurotherapeutic strategies, are greatly needed in order to improve feeding and developmental outcomes. Pulsed pneumatic orocutaneous stimulation has been shown to improve nonnutritive sucking (NNS) skills in preterm infants who exhibit delayed or disordered nipple feeding behaviors. Separately, the study of the salivary transcriptome in neonates has helped identify biomarkers directly linked to successful neonatal oral feeding behavior. The combination of noninvasive treatment strategies and transcriptomic analysis represents an integrative approach to oral feeding in which rapid technological advances and personalized transcriptomics can safely and noninvasively be brought to the bedside to inform medical care decisions and improve care and outcomes.

**Objective:**

The study aimed to conduct a multicenter randomized control trial (RCT) to combine molecular and behavioral methods in an experimental conceptualization approach to map the effects of PULSED somatosensory stimulation on salivary gene expression in the context of the acquisition of oral feeding habits in high-risk human neonates. The aims of this study represent the first attempt to combine noninvasive treatment strategies and transcriptomic assessments of high-risk extremely preterm infants (EPI) to (1) improve oral feeding behavior and skills, (2) further our understanding of the gene ontology of biologically diverse pathways related to oral feeding, (3) use gene expression data to personalize neonatal care and individualize treatment strategies and timing interventions, and (4) improve long-term developmental outcomes.

**Methods:**

A total of 180 extremely preterm infants from three neonatal intensive care units (NICUs) will be randomized to receive either PULSED or SHAM (non-pulsing) orocutaneous intervention simultaneous with tube feedings 3 times per day for 4 weeks, beginning at 30 weeks postconceptional age. Infants will also be assessed 3 times per week for NNS performance, and multiple saliva samples will be obtained each week for transcriptomic analysis, until infants have achieved full oral feeding status. At 18 months corrected age (CA), infants will undergo neurodevelopmental follow-up testing, the results of which will be correlated with feeding outcomes in the neo-and post-natal period and with gene expression data and intervention status.

**Results:**

The ongoing National Institutes of Health funded randomized controlled trial R01HD086088 is actively recruiting participants. The expected completion date of the study is 2021.

**Conclusions:**

Differential salivary gene expression profiles in response to orosensory entrainment intervention are expected to lead to the development of individualized interventions for the diagnosis and management of oral feeding in preterm infants.

**Trial Registration:**

ClinicalTrials.gov NCT02696343; https://clinicaltrials.gov/ct2/show/NCT02696343 (Archived by WebCite at http://www.webcitation.org/6r5NbJ9Ym)

## Introduction

The biological complexities of oral feeding have made it the most advanced neurological milestone of the newborn and a predictor of both short- and long-term developmental outcomes in the at-risk premature neonatal population [1,2]. Successful oral feeing requires the integration of the nervous, craniofacial, gastrointestinal, and respiratory systems, as well as maturation of the sensory systems (vision, hearing, somatosensory, gustatory, and olfactory) and hypothalamic feedback loops of satiety and hunger [3-7]. Given the adverse ex utero environment, the achievement of oral feeding competency is a universal challenge across the extremely premature infant (EPI) population (gestational age<28 weeks). While EPIs represent only a small fraction of those born prematurely (<37 weeks’ gestational age) [8], they are most at risk for significant morbidities (eg, necrotizing enterocolitis, intraventricular hemorrhage, and bronchopulmonary dysplasia) that exacerbate the challenges of learning to feed orally. Due to their compromised respiratory status, EPIs who develop bronchopulmonary dysplasia (BPD) are at greater risk for oral feeding impairments when compared with gestationally-aged (GA) matched infants who do not develop the disease. These infants have been shown to have more difficulty achieving a coordinated suckle-feeding pattern and demonstrate abnormally long periods of deglutition apnea and irregular breathing patterns during feeding [9-14]. These developmental abnormalities place EPIs, specifically those who develop BPD, at increased risk for multiple short- and long-term medical complications. Indeed, more than 40% of children in feeding disorder programs are former preterm infants, strongly linking long-term feeding difficulties to failed oral feeding trials in the NICU [15]. In addition, premature infants who correct to term post-conceptional age (PCA) and who cannot successfully orally feed have been shown to be at increased risk for developmental disabilities [16,17] and may require surgical insertion of a gastrostomy tube to provide adequate enteral nutrition [18].

There is currently no objective and quantitative tool to assess oral feeding maturity in premature infants. The current standard of care to determine oral feeding readiness in the EPI population is to use largely subjective, qualitative, and unvalidated cue-based assessment tools [19-22]. Caregivers can only rely on their clinical acumen and overall physical and developmental assessments to determine when an infant may safely and effectively feed orally. The majority of neonatal intensive care units (NICUs) use assessment tools that allow EPIs who correct to ≥33 weeks’ PCA and who demonstrate appropriate cues with a stable respiratory status to attempt oral feeding trials. These trial-and-error approaches provide no insight into why an infant should fail to orally feed and be unable to assess orocutaneous, neurological, or gastrointestinal developmental stages, particularly at a molecular level. A recent Cochrane Review assessing the benefits of these available feeding assessment tools in reducing hospital length of stay, as well as shortening time to full oral feeds, concluded that “there is no evidence to inform clinical practice” [23]. The review further stated, “research is needed in this area to establish an evidence base for the clinical utility of instruments to assess feeding readiness in the preterm infant population.” The lack of an objective assay to accurately assess feeding skills and behavior in this vulnerable population has not only contributed to neonatal morbidities, such as choking, aspiration, and feeding aversions, but has also resulted in prolonged hospitalizations with average medical costs estimated at US $3,500/day [24,25]. Thus, there is a strong need to advance the field through the integration of novel treatment strategies and objective assessment tools to improve oral feeding skills, while identifying aberrant or delayed maturation of specific developmental systems, which limits feeding success.

A new translational application, approved by the FDA in 2008, has been shown to promote ororhythmic motor patterning (nonnutritive suck) in preterm infants who exhibit delayed or disordered nipple feeding behaviors. This approach is based on mechanosensory entrainment of the suck central pattern generator using servo-controlled pulsed pneumatic stimuli to drive peri- and intra-oral afferents, which in turn modulate local reflex activity and produce nonnutritive suck rhythms [26-31]. Since 2008, this innovative device, known as the NTrainer System, has been improving oral feeding skills in the at-risk premature newborn. However, limited data exist that inform caregivers when to initiate the intervention or identify which infants will positively respond.

To address this need, the current study employs neonatal salivary transcriptomics, a rapidly evolving field that utilizes a safe, easily obtained biofluid for health and systemic disease surveillance. Saliva is a diverse source of genetic material, proteins, metabolites, and microorganisms. Through both extracellular and intracellular trafficking mechanisms in the salivary glands, biomarkers enter into the oral cavity and may be monitored to inform an investigator about an infant’s development, physiology, and pathophysiology [32,33]. In the past decade, it has been found that an enormous amount of neonatal developmental gene expression data is available from as little as 10 *μ* L of saliva [34]. This research has led to the identification of salivary biomarkers that are directly linked to successful neonatal oral feeding behavior [4,35]. However, expression of these genes has only been assessed at two feeding time points: unsuccessful and successful oral feeding. No data currently exist that describe each gene’s expression ontology leading up to oral feeding trials or during the process of oral feeding. Thus, this study aims at generating new and critical gene expression data regarding the developmental milestones required for oral feeding competency in this at-risk population. We hypothesize that some or all of these genes will be positively affected by the PULSED NTrainer intervention and alter their gene expression ontology to expedite oral feeding success. Further, gene expression data may accurately identify those infants who will most benefit from the intervention. Gaining access to this developmental information through noninvasive salivary gland gene expression analyses may allow caregivers to make informed and objective decisions about treatment strategies and optimize timing for the initiation of oral feeding trials.

There is extensive evidence from animal models that support the notion that somatosensory experience delivered to trigeminal mechanosensitive afferents and orofacial motor activity causes (1) structural changes in brain circuitry and (2) modulations in gene expression. The orofacial primary somatosensory cortex (S1) is exquisitely responsive to tactile manipulations of the sensory periphery, making it a classic system for studying plasticity in the developing brain in rodents [36-44] and primates [45]. In rodents, the principal sensory nucleus of the trigeminal nerve (PrV) is primarily responsible for transferring whisker-specific topographical patterning to the contralateral ventroposteromedial nucleus of the thalamus (VPm) [46]. Topographical pattern formation follows a sequential order from Orofacial˃PrV˃VPm˃S1 neocortex in the neonatal period [47]. Early patterning of thalamocortical afferents is dependent on the sensory periphery [39]. Removal of a row of whiskers in the neonatal period results in shrinkage of the layer 4 (L4) cortical areas known as “barrels” assigned to each clipped or extirpated whisker, while adjacent barrels mapped to intact whiskers in S1 expand [48]. This effect is far more robust in the neonatal period than at later ages. Maladaptive stimulation or periods of somatosensory deprivation in the neonatal period affect orofacial map formation in PrV, VPm, and S1 [49,50]. Repetitive somatosensory stimulation or changes in the frequency and strength of activation across synapses can cause physiological changes including long-term potentiation (LTP) or long-term depression (LTD) of neurotransmission [51,52]. These data strongly suggest that there are critical periods during which the presence of specific external or internal conditions is necessary for normal development and that the absence of such conditions leads to irreversible alterations in the organism [53]. To further delineate these critical developmental windows, researchers have begun to focus on identifying differential gene expression before, during, and at the closure of the critical period of plasticity at various levels of the trigeminal pathway [39]. Importantly, recent studies have shown that short- and long-term somatosensory stimulation by enriched environment up-regulates cortical expression of neuropeptide messenger RNAs (mRNA) and down-regulates immediate-early gene mRNAs in the rodent S1 barrel cortex, and they suggest a central role of neuropeptides in tuning S1 circuits by somatosensory experience [54,55]. These gene expression data lay the foundation for this study to integrate somatosensory stimulation and gene expression analysis to improve oral feeding in the human neonate.

An obvious extension of this research will be to assess the potential long-term impact of PULSED NTrainer in the neonatal period on the feeding behavior, growth, and neurodevelopmental outcomes of EPIs at 18 months’ PCA. It is well established that feeding issues may continue long after an EPI is discharged from the hospital and that resultant poor growth and nutrition negatively impacts neurodevelopment [16,17]. Combined with the existing animal data that strongly suggests that there are critical developmental windows for appropriate somatosensory stimulation, we hypothesize that infants exposed to PULSED NTrainer in the neonatal period will have improved growth and neurological outcomes in the first years of life and that gene expression data will be able to specify such “critical periods.”

The purpose of this study is to combine molecular and behavioral methods in an experimental conceptualization approach to map the effects of trigeminal (PULSED orocutaneous) somatosensory stimulation on salivary gene expression in the context of the acquisition of an exquisitely complex oromotor skill, namely oral feeding, in high-risk human neonates. The aims of this study represent the first attempt to combine noninvasive treatment strategies and transcriptomic assessments of high-risk EPIs to (1) improve oral feeding behavior and skills, (2) further our understanding of the gene ontology of biologically diverse pathways related to oral feeding, (3) use gene expression data to personalize neonatal care and individualize treatment strategies and timing interventions, and (4) improve long-term developmental outcomes. This integrative approach to oral feeding is a paradigm shift in neonatal care, where rapidly emerging technological advances and personalized transcriptomics can safely and noninvasively be brought to the neonatal bedside to inform medical care decisions and improve care and outcomes **.**

## Methods

### Participants

Only EPIs born between 24 0/7 and 28 6/7 weeks’ gestation, as determined by obstetric ultrasound at <15 weeks or last menstrual period, are eligible to participate in this study. We will actively enroll EPIs once they have a corrected PCA of ≥29 weeks to limit the number of infants who develop serious sequelae of prematurity and would not be eligible for this study based upon the criteria listed below. Parents of enrolled subjects will receive a Babies R Us $50 gift card for their participation. Subjects will be recruited from three neonatal intensive care units including (1) CHI Health St. Elizabeth (Lincoln, Nebraska), (2) Tufts Medical Center NICU (Boston, Massachusetts), and (3) Santa Clara Valley Medical Center (San Jose, California) by a study site principal investigator (PI) or co-Investigator or the neonatal study coordinator. Informed consent will be obtained before participants’ entry into the study, following consultation with the attending physician and nurse(s). A total of 180 EPIs (approximately equal numbers of males and females, no exclusion based on race or ethnicity) will participate in the study (power >.85, Type I error <.05).

### Exclusion Criteria

EPIs will not be recruited for this study if they have any of the following: (1) chromosomal and congenital anomalies including craniofacial malformation, nervous system anomalies, cyanotic congenital heart disease, gastroschisis, omphalocele, diaphragmatic hernia and other major gastrointestinal anomalies; (2) congenital infection; (3) no documented GA; (4) severe intrauterine growth restriction (IUGR) (3%); (5) abnormal neurological status including head circumference <10th or >90th percentile, intracranial hemorrhage grades III and IV, seizures, meningitis, neurological examination showing abnormal tone or movements of all extremities for PCA; (6) history of necrotizing enterocolitis (stage II and III); and (7) culture-positive sepsis at the time of study enrollment.

### Protocol

**Figure 1:**
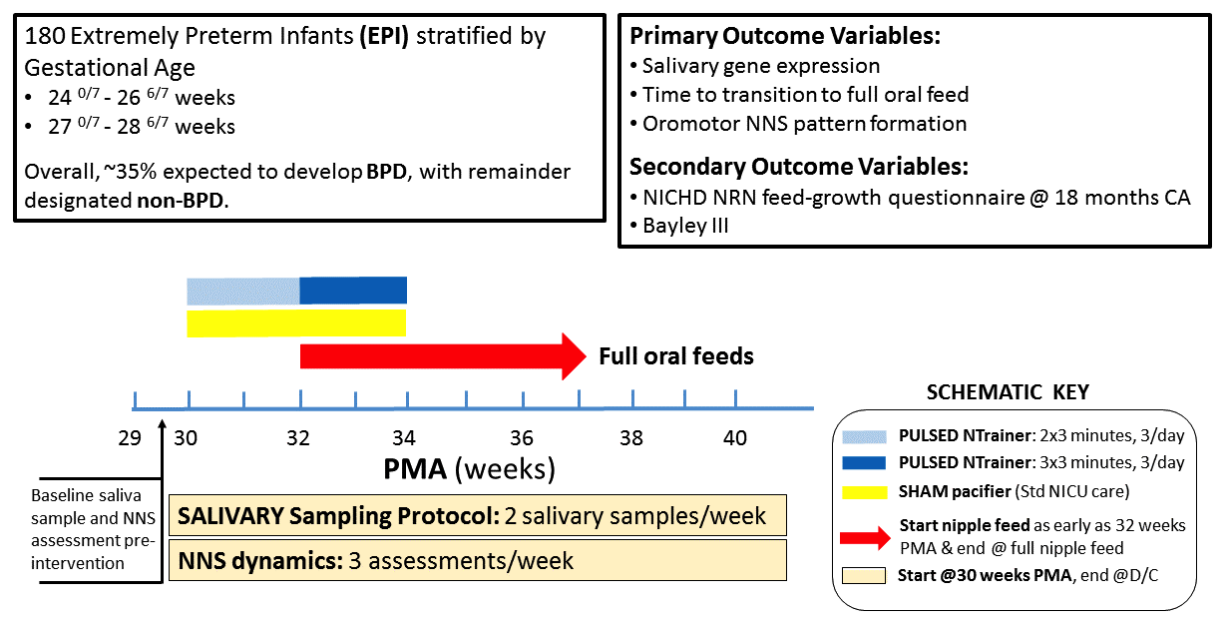
Somatosensory intervention plan for stratified populations of Extremely Premature Infants along with the salivary sampling protocol and digitized measurements of nonnutritive suck progression is shown below, with primary (salivary gene expression, time to attain oral feeds, and nonnutritive suck pattern formation) and secondary (National Institute of Child Human Development Neonatal Research Network feeding-growth questionnaire and Bayley III screener at the neonatal intensive care unit follow-up) outcome variables listed as well.

EPIs will be stratified among two gestational age groups (24 0/7-26 6/7 weeks and 27 0/7-28 6/7 weeks). Each infant will be randomized to receive either the PULSED NTrainer or SHAM intervention using a software random integer function in Minitab version 17. As shown in Figure 1, infants assigned to the PULSED NTrainer group will receive a progressive dose of the pulsatile orocutaneous stimulation. Beginning at 30 weeks’ PCA, these infants will receive 2 weeks of low-dose PULSED NTrainer stimulation (2 x 3-minute blocks) with a 1-minute stimulus “off-period” between the stimulation blocks. This form of stimulation will be given simultaneously with tube feedings 3 times/day. The stimulus dose will then be increased over the next 2 weeks (3 x 3-minute blocks of PULSED NTrainer stimulation) with a 1-minute stimulus “off-period” between the stimulation blocks, also given simultaneously with tube feedings 3 times/day. EPIs randomized to the SHAM condition will be given a regular silicone pacifier during tube feedings over the same time period, and will be handled in exactly the same way as those infants in the experimental group of the study, with the exception of the PULSED inputs from the pacifier. To ensure blinding, only study site PIs or co-Investigators and the neonatal study coordinators will be informed of infants’ group assignments. Physicians, nurses, and other NICU care staff will not be informed about the infants assigned to the intervention group.

### Orocutaneous Stimulation Regimen

**Figure 2:**
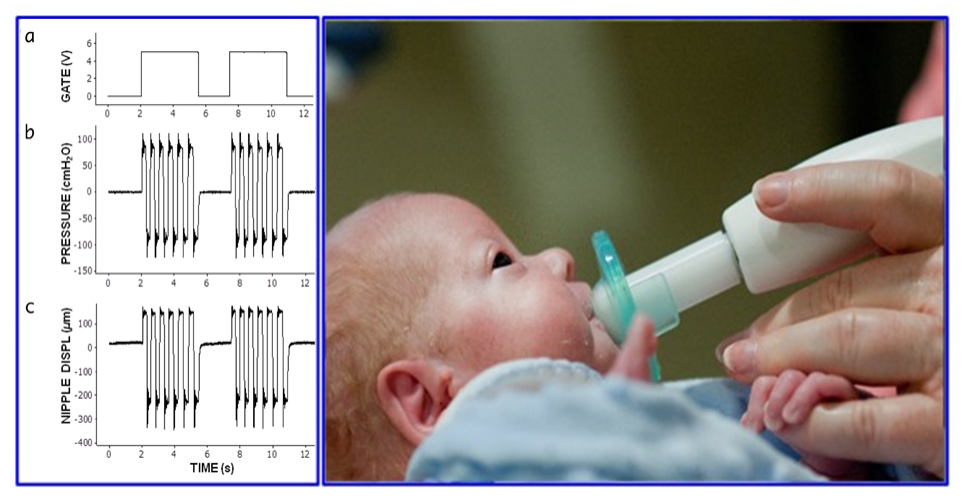
Preterm infant receiving PULSED NTrainer stimulation during gavage feeding in the neonatal intensive care unit, with a nasogastric tube placed through the left nares (not visible); pneumatic stimulus control signals and output through the pacifier nipple are shown in the left panel: (a) voltage-controller gate signal, (b) intraluminal pressure (inside) the nipple, and (c) mechanical displacement at the nipple cylinder wall (Photo courtesy of Innara Health, Inc., Olathe, Kansas USA).

The NTrainer PULSED orocutaneous stimulus consists of a series of 6-cycle bursts that are delivered by a servo-controlled pneumatic amplifier (NTrainer System) to the lumen of a standard silicone pacifier (eg, WeeSoothie or Soothie). These pneumatic bursts are frequency modulated (FM) from 2.8 to 1.6 Hz across the 6-cycle structure, with a 2-second pause period between bursts (Figure 2). Individual pressure cycles have a 31 millisecond (ms) rise or fall time to ensure salient stimulus spectra with significant energy from DC-16 Hz [56]. Frequency modulation is a physiologic feature of the nonnutritive suck (NNS) in preterm infants [57]. A total of 34 bursts are presented in a 3-minute block. A 1-minute rest period (no stimulation) occurs between stimulation blocks. Criteria for initiation of orocutaneous therapy include the following: (1) stable vital signs and not on continuous vasopressor medications, (2) tolerating enteral feeds in previous 48 hours, and (3) not intubated and mechanically ventilated. If the infant is on nasal intermittent positive pressure ventilation, continuous positive airway pressure or nasal cannula >2 liters per minute, then the fraction of inspired oxygen (FiO2) must be <40%.

### Nonnutritive Suck Assessment and Automated Nonnutritive Suck Digital Signal Processing and Feature Extraction

In addition to the oral stimulation interventions (PULSED NTrainer vs SHAM pacifier), all study infants will be assessed 3 times/week (eg, Monday/Wednesday/Friday) for NNS performance. The NTrainer System will be used in “ *assessment mode* ” to record the compression dynamics of NNS during a 3-minute session to immediately precede a tube feeding that is not associated with an intervention condition.

The most active 2-minute period of NNS behavior based on suck cycle count is automatically extracted from each suck assessment data file using an automated waveform feature extraction algorithm on the NTrainer System. The NNS pressure waveform is band-pass filtered (0.5-20 Hz) to remove low frequency offsets due to tongue or jaw posturing and thermal drift associated with oral contact on the pacifier bulb and to remove high-frequency jitter. Pressure peaks >1.6 cm H_2_O are subjected to feature extraction criteria, including suck cycle symmetry, cycle duration, and burst identification defined as two or more NNS events occurring within 1200 ms. This algorithm permits objective identification of NNS burst activity distinct from non-NNS mouthing compressions or tongue thrusts against the pacifier. Four measures will be objectively extracted from the indexed records of suck compression, including minute-rates for (1) NNS bursts where an individual burst includes 2 or more suck cycles, (2) NNS cycle events defined as suck compression cycles with cycle periods less than 1200 ms, (3) Total oral compressions defined as the sum of all pressure events, and (4) NNS compression pressure expressed in cm H_2_O [56-63].

### Per os (PO) Feeding Advancement

EPIs will advance on a standardized cue-based feeding schedule utilized by each site NICU, known as Infant Driven Feeding [64,65] that will lead to full nipple feeds. This standardization of oral feeding advancements across sites will limit confounders that may ultimately skew the data.

EPI participant data will be managed with our Neonatal Oromotor Database, custom software developed in the Barlow laboratory specifically for NTrainer studies in the NICU. This database software is compatible with MS WIN 7 8.2, and 10 operating systems and is password-protected and coded as executable for MS ACCESS 2013. This software provides a paperless, efficient system for NICU study personnel to log daily information including GA, growth parameters, medications, oxygen requirement, feeding history, medical procedure log, comments, as well as NTrainer or SHAM and salivary sampling dates on enrolled infants.

### Saliva Collection, Processing, and Gene Expression Analysis

#### Saliva Collection

Saliva samples will be collected from all enrolled infants on the first day, before receiving either the SHAM or PULSED NTrainer intervention. This sample will serve as our baseline gene expression profile. Samples will then be obtained twice per week (~ 3 day intervals) up to the time of achievement of full oral feeds or discharge from the hospital with either a nasogastric tube or gastronomy tube.

Saliva samples at each site will be collected with techniques that have been optimized and validated through the Maron laboratory [66]. Briefly, saliva samples will be collected with a 1 mL syringe, end caps removed and attached to low wall suction. Saliva will be collected for approximately 20 seconds and immediately stabilized in 500 μL of Qiagen’s RNA Protect Saliva to halt gene expression changes, inhibit destructive ribonucleases (RNases), and limit microbial overgrowth. Samples will be vortexed briefly and frozen at −20°C. Once frozen, samples are stable for up to 18 months before the need for total ribonucleic acid (RNA) extraction. Thus, samples from Nebraska and Santa Clara will be shipped once a month on ice overnight to the Maron laboratory at the Mother Infant Research Institute at Tufts Medical Center in Boston, Massachusetts, for processing.

#### Generation of a Saliva Biobank

One additional saliva sample will be obtained each week from all enrolled subjects to generate a biobank repository. The rationale for this approach is twofold. First, it is possible that during shipment or processing, salivary RNA may be destroyed and not be of sufficient quality to incorporate into the study. Prior studies from this lab have demonstrated a 10% failure rate of gene amplification in saliva samples [4]. Banking an additional sample each week from study subjects will ensure that we will limit loss of data points. Second, we anticipate that there are informative salivary biomarkers of oral feeding maturation that have yet to be identified. By generating a biobank of additional saliva samples that may be retrospectively interrogated on either a microarray gene expression platform or with RNA sequencing, we will have the potential to discover novel hypotheses about gene-gene interactions and/or transcriptional regulatory processes related to oral feeding in the EPI population.

#### Total Ribonucleic Acid Extraction

Salivary samples will extracted for total RNA using established protocols optimized in the Maron laboratory [66]. RNA extraction will occur with the QIAGEN RNAProtect Saliva Mini kit, per manufacturer’s instructions. On column DNase treatment will be performed for each sample to eliminate genomic DNA contamination. Samples that are part of the biobank will be frozen at −80° C, pending need for future analysis. Samples that will be used for quantitative reverse transcription polymerase chain reaction (RT-qPCR) experiments will first undergo complementary deoxyribonucleic acid (cDNA) conversion with the Life Technologies SuperScript Vilo kit, per manufacturer’s instructions. cDNA will be stored at −80°C in the Mother Infant Research Institute at Tufts Medical Center, pending gene expression analysis.

#### Gene Expression Analysis

For the purpose of this study, we will interrogate each sample for nine genes, six target genes of interest and three reference genes for quality control and potential normalization of gene expression data. Genes to be analyzed in this study have been previously shown to be directly linked to oral feeding in the newborn and include *PLXNA1*, *PLXNA3*, *NPY2R*, *WNT3*, *AMPK*, and *NPHP4*. The three reference genes include *GAPDH*, *HPRT1*, and *YWHAZ*, all of which have been shown to maintain stable gene expression across advancing PCA [67]. All cDNA samples will undergo targeted preamplification with a custom TaqMan PreAmp Master Mix for all nine genes. This targeted approach to amplification will ensure that only those genes of interest will be amplified and not all genes across the transcriptome that may introduce bias. Preamplified cDNA will then undergo PCR with TaqMan Fast Advance Master Mix on the Life Technologies Quant Studio 7 Flex Real-Time PCR System. This instrument is housed at the Mother Infant Research Institute at Tufts Medical Center and is an advanced state-of-the-art polymerase chain reaction (PCR) machine and software system. All efforts will be made to adhere to the Minimum Information for Publication of Quantitative Real-Time PCR Experiments (MIQE) guidelines for this study [68]. All samples will be run in duplicate with appropriate positive and negative controls.

#### Initial Gene Expression Analyses

All gene expression data will be normalized on the Quant Studio 7 Flex Real-Time PCR System. Only samples that have successful amplification of all three reference genes will be considered in the analysis. Previously, genes have been informative in a binary fashion (+/- expression) [4]. However, for the purpose of this study, we will be prepared to calculate relative gene expression of each target gene with the delta delta cycle threshold (ΔΔCt) method [69]. In accordance to the MIQE guidelines, we will use the three reference genes for normalization and determine a geometric mean of their Ct in each sample to calculate relative gene expression [66]. Normalized data will then be given to our statistician and bioinformatic collaborator for analysis.

### Neurodevelopmental Follow-Up

Each center site in this study has a Neonatal Follow-Up Clinic that performs neurodevelopment testing on EPIs from discharge up to three years of life by certified examiners as part of standard of care. Thus, developmental follow-up data will be available from all study subjects, regardless of intervention and free of charge. As part of standard of care, each site will complete the Bayley Scales of Infant and Toddler Development 3rd Edition [70] on EPIs at 18 to 24 months’ corrected age. The primary subtests of interest include *Fine Motor* (prehension, motor speed and planning, perceptual-motor integration, reaching, grasping, and object manipulation), *Gross Motor* (dynamic movement, including walking, jumping, running, stairs, and so on; motor planning; balance; and perceptual-motor integration), *Cognition* (puzzle assembly, object completion, means-ends manipulation, representational play, counting, and matching colors), and *Language* (receptive and expressive language). These data will be recorded, analyzed, and correlated with feeding outcomes in the neo-and post-natal period and with gene expression data and intervention status. In addition, growth parameters including head circumference, length, and weight will be recorded.

Feeding behavior plays a significant role in neurodevelopmental outcomes. Recent findings from the NICHD Neonatal Research Network (NRN) indicated that at 18 months’ CA, premature infants with a history of feeding difficulties are more likely to have language delay. Neuromotor status and days on mechanical ventilation are important risk factors associated with these outcomes [71]. A follow-up of feeding status will be completed when our study infants reach 18 months’ CA, using the NICHD NRN 18-month Feeding-Growth-Nutrition Questionnaire. This questionnaire includes a simple checklist format about medical history since the NICU (re-hospitalization, primary cause, time period, LOS, and time in ICU), medications, seizures, supplemental oxygen and respiratory monitoring, oromotor skills (independent feeds, dependent oral feeds, limited oral feeding, and no oral feeding), nasogastric (NG) or total parenteral nutrition (TPN) feeds, feeding behaviors (aversion and swallowing-dysphagia), aspiration (food down windpipe or choking), spit-ups, high calorie supplements, oral diet texture (thin vs thickened liquids, soft solids, and table food), and surgical operations. Completion time by the parent is 15 minutes or less. The feeding questionnaire and a pre-posted return envelope will be mailed to the parent when a given study infant attains 17.5 months of age. A cover letter will accompany the 3-page questionnaire explaining that a nurse or study specialist from this research project will be available to assist with the questionnaire.

### Statistical Analysis

To address the relationship between PULSED NTrainer stimulation and salivary gene expression profiles and duration to full oral feeds, descriptive statistics and bivariate tests will examine the associations of gene expressions with successful oral feeding at each measurement time point. Diagnostic performance statistics, such as sensitivity, specificity, positive predictive value, and negative predictive value, will also be computed for each selected gene. Frailty modeling techniques will compare the time to full oral feed between SHAM and PULSED NTrainer treatments. Given the clustered longitudinal structure of our data (ie, EPIs at three NICUs are observed repeatedly over a 6- to 8-week period), a mixed modeling approach will be used to properly account for dependency among observations. Specifically, general or generalized mixed models will estimate overall group difference between SHAM and PULSED NTrainer treatments (group effect), developmental pattern over time (time effect), and group difference in this pattern (time-by-group interaction) separately for gene expressions, NNS formation, and oral feeding skills. A significant group effect and/or time-by-group interaction will indicate significant intervention effect. Models will include GA, PCA, sex, and antenatal steroids as covariates, thereby providing unbiased estimates of intervention effects. In subsequent mixed modeling of feeding status as a dependent variable, we will calculate the odds ratio (OR) and 95% CI for successful oral feeding in association with the expression of each gene. Grids of estimated probabilities of full oral feeding for different combinations of gene expressions, NTrainer treatments, GA, PCA, and sex will be constructed. In secondary analyses, the proposed statistical analyses will be conducted separately among BPD and non-BPD EPIs. All analyses will be conducted using R and SAS 9.4 [72].

To address the power and accuracy of predicting oral feeding status with the use of feeding-readiness gene expression profiles and NTrainer treatments, generalized linear models with a logit link (ie, logistic regression) will be evaluated separately for different infant subgroups of GA, PCA, sex, and BPD status. For example, the coefficient of determination (*R*^2^) and area under the curve (AUC) estimated at each measurement time point will suggest the presence and timing of critical periods when oral somatosensory stimulation may enhance or inhibit oral feeding attainment. Also, learned Bayesian networks will be applied to the gene expression data to identify informative gene clusters that discriminate between neonates who received PULSED NTrainer and those who received SHAM. We will further differentiate subjects for whom PULSED NTrainer was an effective intervention to improve feeding outcomes and for those in whom it was not [73]. Data analysis will be performed in a three stage sequence including data preparation, selection of predictive gene clusters, and Bayesian network probabilistic analysis. Data will first be organized into two datasets: one that includes all gene expression data and one that includes gene expression data from the last sample obtained (LSO) at the end of the intervention. Six datasets will then be generated: (1) all screening observations; (2) all data from LSO; (3) differenced observations from SHAM, responders, and nonresponders; (4) differenced nonresponders and SHAM; (5) differenced nonresponders and responders; and (6) differenced responders and SHAM. The “all screening observation” dataset will be used as a baseline measure for the other five partitions in the predictive models.

Bayesian network structure and parameter estimates will be learned directly from study generated data partitions listed above (1-6) [74]. The program will assume an equal likelihood for all models, sequentially searching the set of all models and then assigning a network score based on either Bayes Factor (BF) or Akaike Information Criterion (AIC) criteria. A heuristic search algorithm (K2 algorithm) will be used to traverse the model space [75]. As both gene expression levels (absolute or binary [+/- gene expression]) and changes in gene expression (differenced) may be predictive of response to the PULSED NTrainer intervention, we will consider both in our analysis. To identify which gene ontological profiles were critical to the predictive value of the Bayesian network models, the gene clusters found in the baseline screening data will be mapped on the LSO Bayesian network using the BF score type. We will examine the importance of these genes’ expression ontology profiles in defining responders and nonresponders by re-evaluating network prediction accuracy.

In order to mathematically model both the transition time-to-oral feeds and salivary gene expression profiles with their predictive relationships to infant feeding, growth, and neurodevelopmental outcomes at 18 months’ CA, general mixed models (ie, individual growth models) will be used to identify the developmental pattern of change (time effect) in NNS formation, salivary gene expression profiles, and oral feeding skills. The change pattern will be compared between SHAM and PULSED NTrainer treatments, BPD conditions, and their combinations as interactions with the time effect in the models. Then, these data will be combined with the NICHD NRN feed-growth-nutrition data and Bayley III (raw and standardized) scores of cognitive, motor, and language skills at 18 months’ CA. General linear models (ie, regression) will examine the predictive power of oral feeding and NNS oromotor skills at the exit of the NICU on the feeding, growth, and Bayley III subtest markers at 18 months’ CA. In separate models, these outcomes will be compared between SHAM and PULSED NTrainer treatments (ie, multivariate analysis of covariance). All models will include GA and sex as covariates.

### Sample Size and Power

This study will utilize a stratified random sampling method, where 180 EPIs stratified by GA (24 0/7-26 6/7 weeks or 27 0/7-28 6/7 weeks) are randomly assigned to SHAM or PULSED NTrainer treatment within each GA stratum. Of the 180 EPIs, we expect 35.0% (63/180) will develop BPD. This estimate is based on an evaluation of the incidence of BPD among EPIs at the 3 participating NICUs. The total sample size of 180 has been determined in order to achieve adequate statistical power in evaluating the effectiveness of NTrainer stimulation. With regard to the analyses on gene expression profiles, we estimated effect sizes corresponding to the treatment group differences in diagnosis accuracy for the most predictive genes of feeding success. Anticipating that SHAM infants will show sensitivity and specificity levels comparable to the values observed in Maron et al [4] and that PULSED infants will provide greater levels of sensitivity and specificity (an increase by 0.10; ie, enhanced expression of those genes), the estimated effect sizes are in the small to medium range (Cohen *f*=0.10-0.19; see Table 1). Given these estimates, a sample of 180 infants is expected to provide, on average, 82-85% power (range=78-96%) to compare the predictive associations of gene expressions with oral feeding status, even under a high attrition rate of 20%.

Our recent NTrainer trial (NIH DC003311) with preterm infants [56,59] also indicated moderate effects on the primary NNS oromotor measures (*f*=0.22-0.26; see Table 2). Power analysis based on this empirical data reveals that the analyses on NNS formation and oral feeding skills will have, on average, 96% power (range=95-98%), with the sample size of 180 and 20% attrition rate. We will error on the conservative side of our power calculation and, therefore, recruit a total of 180 infants, which is expected to yield 63 BPD and 117 non-BPD EPIs. After 20% attrition, the net number of EPIs is 50 BPD and 94 non-BPD.

**Table 1:** Effect size and power for genetics measures.

Gene	Sensitivity^a^	*f*	Power	Specificity^a^	*f*	Power
*PLXNA1*	0.85	0.17	0.93	0.23	0.11	0.81
*AMPK*	0.96	0.19	0.96	0.08	0.15	0.90
*WNT3*	0.17	0.12	0.83	0.72	0.12	0.83
*NPY2R*	0.39	0.10	0.78	0.53	0.10	0.78
*NPHP4*	0.58	0.10	0.78	0.35	0.10	0.78
Average		0.14	0.85		0.12	0.82

^a^values from Maron et al [4].

**Table 2:** Effect size and power for nonnutritive suck oromotor measures.

Variable	SHAM^a^, mean (SD)	PULSED^a^, mean (SD)	*f*	Power
NNS^b^ Bursts/minute	7.85 (2.36)	8.79 (1.93)	0.22	0.95
NNS^b^ events/minute	64.67 (22.18)	74.92 (20.37)	0.24	0.96
Total oral compressions/minute	71.02 (24.66)	83.75 (23.67)	0.26	0.98
Average			0.24	0.96

^a^Values from Barlow et al.

^b^NNS: Nonnutritive suck.

## Results

This ongoing National Institutes of Health (NIH) funded research is being conducted at three NICUs across the United States: (1) CHI Health St. Elizabeth (Lincoln, Nebraska), 2) Tufts Medical Center (Boston, Massachusetts)], and (3) Santa Clara Valley Medical Center (San Jose, California). The research protocol has been approved by each site’s Institutional Review Board (IRB). Participant recruitment began in July 2016, and NTrainer intervention is expected to be completed by 2020. Neurodevelopmental follow-up testing will begin in 2018.

The primary outcome variables include salivary gene expression of a panel of genes previously identified as playing a role in oral feeding of preterm infants, time to transition to full oral feeding, and oromotor NNS pattern formation. Secondary outcome variables include: NICHD NRN 18-month Feeding-Growth-Nutrition Questionnaire, and Bayley III scores of cognitive, motor, and language skills at 18 months’ corrected age (CA).

## Discussion

Oral feeding is a complex neurological milestone that can pose a significant challenge for many preterm infants, particularly for those born extremely prematurely (<28 weeks gestational age), as well as those with a compromised respiratory status. Persistent feeding difficulties can result in numerous short- and long-term medical complications, as well as place infants at risk for developmental disabilities. Currently, there is no objective method for treating delayed or disordered feeding skills, or for assessing oral feeding maturity. Therefore, there is a strong need for objective assessment tools and novel therapeutic interventions to identify the underlying mechanisms that delay and disrupt oral feeding success and provide evidence-based intervention strategies to help ameliorate outcomes. The current study aims to combine safe, noninvasive treatment strategies and salivary diagnostics in EPIs to improve oral feeding skills as well as long-term neurodevelopmental outcomes and to further our understanding of the gene ontology of biological pathways related to oral feeding, which may ultimately individualize the type and timing of interventions and personalize neonatal care.

## Appendix

Multimedia Appendix 1 NIH summary statement - study section peer review.

## Abbreviations

BPD: bronchopulmonary dysplasia
CA: corrected age
cDNA: complementary deoxyribonucleic acid
EPI: extremely preterm infant
FiO2: fraction of inspired oxygen
IUGR: intrauterine growth restriction
mRNA: messenger RNA
National Institute of Child Human Development: NICHD
National Institutes of Health: NIH
NG: nasogastric
NNS: nonnutritive suck
NRN: neonatal research network
PCR: polymerase chain reaction
PI: principal investigator
PMA: postmenstrual age
PO: per os
RNA: ribonucleic acid
RNase: ribonuclease
RT-qPCR: quantitative reverse transcription polymerase chain reaction
TPN: total parenteral nutrition
