# Supplementary material for: Somatosensory Modulation of Salivary Gene Expression and Oral Feeding in Preterm Infants: Randomized Controlled Trial
Source: JMIR Res Protoc. 2017 Jun 14;6(6):e113. doi: 10.2196/resprot.7712 (PMC5489710; doi:10.2196/resprot.7712)
Supplement: Multimedia Appendix 1 [file resprot_v6i6e113_app1.PDF]

SUMMARY STATEMENT
(Privileged Communication)

PROGRAM CONTACT:
DANIEL RAITEN
(301) 435-7568
raitend@mail.nih.gov

Release Date: **08/26/2015**

Application Number: **1 R01 HD086088-01**

Principal Investigators (Listed Alphabetically):
BARLOW, STEVEN M PHD (Contact)
MARON, JILL LAMANNA MD

Applicant Organization: UNIVERSITY OF NEBRASKA LINCOLN

Review Group: **CHHD-H**
Biobehavioral and Behavioral Sciences Subcommittee

Meeting Date: **06/10/2015** ***RFA/PA:*** **PA13-302**
Council: **OCT 2015** ***PCC:*** **PGNB -DR**
Requested Start: **09/01/2015**

Project Title: **Somatosensory Modulation of Salivary Gene Expression and Oral Feeding in Preterm Infants**
SRG Action: **Impact Score: 23 Percentile: 9 #**
Next Steps: **Visit http://grants.nih.gov/grants/next_steps.htm**
Human Subjects: 30-Human subjects involved - Certified, no SRG concerns
Animal Subjects: 10-No live vertebrate animals involved for competing appl.
Gender: **1A-Both genders, scientifically acceptable**
Minority: **1A-Minorities and non-minorities, scientifically acceptable**
Children: **1A-Both Children and Adults, scientifically acceptable**
Clinical Research - not NIH-defined Phase III Trial

Project Year	Direct Costs Requested	Estimated Total Cost
1	499,436	720,438
2	452,034	652,061
3	481,656	694,790
4	499,266	720,193
5	491,716	709,302
TOTAL	2,424,108	3,496,784

ADMINISTRATIVE BUDGET NOTE: The budget shown is the requested budget and has not been adjusted to reflect any recommendations made by reviewers. If an award is planned, the costs will be calculated by Institute grants management staff based on the recommendations outlined below in the COMMITTEE BUDGET RECOMMENDATIONS section.

1R01HD086088-01 BARLOW, STEVEN

RESUME AND SUMMARY OF DISCUSSION: This is a new R01 application by Dr. Barlow from the University of Nebraska/Lincoln that proposes to develop a personalized technique for assessing and treating feeding difficulties in extremely preterm infants and to test transcriptomics as a predictor of readiness and outcome. ThIs multicenter trial compares the use of a pacifier that stimulates and measures sucking in an individualized manner with a sham stimulation. The project is headed by a strong investigative team with good integration of their technological and treatment interventions. The proposed research is highly innovative and potentially of high impact. Despite the reviewers' high enthusiasm for the project, several important concerns were noted. No data are to be collected on families and thus, important moderating variables are ignored. A set of genes are targeted, but the applicant does not propose sufficient structural genetic and epigenetic analyses needed to understand expression effects. The sham comparison may not be extensive enough to ensure blinding of caregivers. The direct behavioral measures are not comprehensive and the Bayley will not provide sufficiently rich data. It is unclear if the PIs, Dr. Barlow and Dr. Maron, are experienced in leading multisite clinical trials. Overall, this application was considered to be highly innovative and to have great potential to contribute to an important area of science.

DESCRIPTION (provided by applicant): Extremely preterm infants (EPIs) (< 28 weeks' gestation) have increased susceptibility for multiple significant short- and long-term medical complications that significantly delay the development of essential milestones, such as oral feeding, required for discharge. Further, infants who develop bronchopulmonary dysplasia (BPD) are at even greater risk for oral feeding difficulties compared to gestationally aged matched controls. Despite the frequency of oral feeding difficulties in this at-risk population, there currently exists no objective assessment tool to predict when an infant may safely feed by mouth and only limited intervention strategies to improve feeding behavior. The overall goal of this research proposal is to pair the expertise of Co-PIs Drs. Steven Barlow and Jill Maron to develop an innovative, novel, personalized and integrative approach that directly addresses the important need for oral feeding treatment strategies for the EPI population. In this multicenter trial, 180 EPIs will be enrolled from three neonatal intensive care units (CHI Health St. Elizabeth, Lincoln, NE; Tufts Medical Center, Boston, MA; and Santa Clara Valley Medical Center, Santa Jose, CA). Infants will be randomized to receive either PULSED orocutaneous somatosensory stimulation (PULSED NTrainer®) previously developed in the Barlow Laboratory, or SHAM (blind pacifier) starting at 30 weeks' post- conceptional age (PCA) up to the achievement of full oral feeds or discharge home. Serial salivary samples (n=2/week) will be obtained concomitantly to examine the gene expression profiles of six biomarkers (AMPK, NPY2R, NPHP4, WNT3, PLXNA1, PLXNA3) previously shown by the Maron Laboratory to be associated with oral feeding success in the premature newborn. In Aim #1, we will test the hypothesis that infants who received PULSED NTrainer® intervention will have a shortened duration of time to achieve full oral feeds and that their salivary gene expression profiles will correlate with feeding success. Infants will be stratified based upon their gestational age, sex, and BPD status. In Aim #2, computational analyses of both clinical and gene expression data from Aim #1 will be performed to identify responders and non-responders to the PULSED NTrainer® intervention and to identify critical times in neurodevelopment when infants may most benefit from the intervention. Finally in Aim #3, infants will undergo Bayley III Developmental Screen at 18-24 months' PCA to assess both the long-term effect of the PULSED NTrainer® on feeding behavior and neurodevelopment and the accuracy of neonatal salivary gene expression profiles to predict impairments later in childhood. Infants will also be assessed with the NICHD NRN 18-month Feeding-Growth-Nutrition Questionnaire to further our understanding of the PULSED NTrainer® on long-term growth and feeding behavior.

PUBLIC HEALTH RELEVANCE: Two innovative approaches, pulsatile orocutaneous entrainment of non-nutritive suck via NTrainer® device technology and serial salivary gene expression analyses, will be merged to examine the relation between gene expression, oral somatosensory stimulation, feeding behavior, and neurodevelopmental outcomes at 18 months corrected age (CA) on 180 extremely preterm infants [EPIs] (< 28 weeks GA) enrolled at three neonatal intensive care units: CHI Health St. Elizabeth (Lincoln, NE), Tufts Medical Center (Boston, MA), and Santa Clara Valley Medical Center (San Jose, CA). EPIs will be randomized to SHAM (blind pacifier) or PULSED NTrainer treatment groups, and stratified by GA, sex, and bronchopulmonary dysplasia status (BPD vs non-BPD). We hypothesize that the combination of the NTrainer® intervention for improved oral feeding skills, along with objective salivary gene expression data to monitor response to treatment and feeding development, will result in a novel, objective, and personalized approach to neonatal oral feeding and reduce the duration of time to attain oral feeds while improving feeding, growth and neurodevelopmental outcomes at 18 months' CA.

CRITIQUE NOTE: The sections that follow are the essentially unedited, verbatim comments of the reviewers assigned to this application. They are provided to illustrate the range of opinions expressed. The application was discussed and scored by all reviewers present. The attached commentaries may not necessarily reflect the position of the reviewers at the close of group discussion, nor the final majority opinion of the group. The Resume and Summary of Discussion, however, is the authoritative representation of the final outcome of the group discussion.

CRITIQUE 1:

Significance: 1
Investigator(s): 1
Innovation: 1
Approach: 4
Environment: 1

Overall Impact: This application is to develop an innovative, novel, personalized and integrative approach that directly addresses the important need for oral feeding treatment strategies for the extremely preterm infant (EPI) population. In this multicenter trial, 180 EPIs will be enrolled from three neonatal intensive care units (CHI Health St. Elizabeth, Lincoln, NE; Tufts Medical Center, Boston, MA; and Santa Clara Valley Medical Center, Santa Jose, CA). Infants will be randomized to receive either PULSED orocutaneous somatosensory stimulation (PULSED NTrainer®) previously developed in the Barlow Laboratory, or SHAM (blind pacifier) starting at 30 weeks' postconceptional age (PCA) up to the achievement of full oral feeds or discharge home. Serial salivary samples (n=2/week) will be obtained concomitantly to examine the gene expression profiles of six biomarkers (*AMPK*, *NPY2R*, *NPHP4*, *WNT3*, *PLXNA1*, *PLXNA3*) previously shown by the Maron Laboratory to be associated with oral feeding success in the premature newborn. In Aim #1, the hypothesis that infants who receive the PULSED NTrainer® intervention will have a shortened duration of time to achieve full oral feeds and that their salivary gene expression profiles will correlate with feeding success. Infants will be stratified based upon their gestational age, sex, and BPD status. In Aim #2, computational analyses of both clinical and gene expression data from Aim #1 will be performed to identify responders and non-responders to the PULSED NTrainer® intervention and to identify critical times in neurodevelopment when infants may most benefit from the intervention. Finally in Aim #3, infants will undergo Bayley III Developmental Screen at 18-24 months' PCA to assess both the long-term effect of the PULSED NTrainer® on feeding behavior and neurodevelopment and the accuracy of neonatal salivary gene expression profiles to predict impairments later in childhood. Infants will also be assessed with the NICHD NRN 18-month Feeding-Growth-Nutrition Questionnaire to further the field's understanding of

the PULSED NTrainer® on long-term growth and feeding behavior. The study has the potential to be of high impact.

1. Significance:

Strengths

- As prematurity is an important public-health problem, the study is highly impactful in its potential.

Weaknesses

- None.

2. Investigator(s):

Strengths

- The team of investigators is outstanding.

Weaknesses

- None.

3. Innovation:

Strengths

- The study is highly innovative in its capacity to push the boundaries of translational science.

Weaknesses

- None.

4. Approach:

Strengths

- It is a well-designed study; only minor weaknesses are noticed.

Weaknesses

- Parents (and mothers in particular) are important elements in caring for prematurely born infants. It seems that it is important to include some characteristics of parenting/family dynamics (as modulating variables) in predicting the outcomes of any intervention.
- Multiple levels of genomic analyses (not only expression, but also epigenetic and structural variation analyses) should be considered.
- The Aim 3 outcomes are not sufficiently sophisticated/maximizing the outcome of the intervention (given what the field knows about sucking/feeding, perhaps language outcome should be considered particularly carefully).

5. Environment:

Strengths

- Excellent.

Weaknesses

- No concerns.

Protections for Human Subjects:

Acceptable Risks and/or Adequate Protections

- The protocol is well designed.

Data and Safety Monitoring Plan (Applicable for Clinical Trials Only):

- Not Applicable (No Clinical Trials)

Inclusion of Women, Minorities and Children and not IRB Exemption #4.

- Sex/Gender: Distribution justified scientifically
- Race/Ethnicity: Distribution justified scientifically
- Inclusion/Exclusion of Children under 21: Including ages < 21 justified scientifically
- The demographic structure of the sample is adequate.

Vertebrate Animals:

- Not Applicable (No Vertebrate Animals)

Biohazards:

- Not Applicable (No Biohazards)

Resource Sharing Plans:

- Acceptable

Budget and Period of Support:

- Recommend as Requested

CRITIQUE 2:

Significance: 2
Investigator(s): 2
Innovation: 1
Approach: 4
Environment: 2

Overall Impact: A particularly innovative proposal that tests the utility of a device-based method for improving oral feeding in extremely premature infants, and to test transcriptomics as a predictor of readiness and outcome. This multi-site clinical trial involves multiple investigators with diverse expertise and experience to support the study, though it lacks an experienced, central leader for the clinical trial execution. The proposal is also somewhat neglectful of the possibility that a direct clinical

measure of sucking, which will be collected, might be employed as a predictor of feeding readiness, favoring instead the more distal transcriptomic measure. The study's plan to involve 24- and 25-week preemies extends beyond the preliminary data, without a specific case being made for this extension. In addition, the control (sham) condition may not be fully adequate. The mechanism for central (vs. site-based) safety monitoring is not explicitly described.

1. Significance:

Strengths

- The development of feeding abilities is one of the most important tasks faced by all infants, and is commonly compromised among those born premature. As the applicants note, there are no objective methods for determining which infants are ready for oral feeding, and certainly no evidence-based methods for promoting such readiness. As such, the significance of the proposed work is very high.

Weaknesses

- It is unclear why transcriptomic measures are hypothesized to be potentially valuable predictors of readiness for feeding intervention or feeding readiness, when direct measures of oromotor function (such as the applicants' own measure of sucking) are available and potentially more appropriate in the clinical context (rapidity of testing and lower cost).
- The applicants do not explain why they focus exclusive on extremely premature infants, when other premature infants also are at risk, and this work would also be significant for that population.

2. Investigator(s):

Strengths

- PI Barlow has a distinguished record of accomplishment in developing the NTrainer device and in many other biomedical device applications for assessment of sensorimotor behaviors and control.
- MPI Maron is a leader in research on neonatal non-invasive transcriptomics and has highly relevant experience in identifying transcriptomic correlates of feeding behavior in neonates.
- Dr. Govindaswami brings strong experience in clinical trials, including previous collaboration with Drs. Barlow & Song. Dr. Jegatheesen also has strong prior history in clinical trials and with Barlow & Song.
- The statistician Prof. Lee is at a 4th institution, but has a good record of collaboration with the PI.

Weaknesses

- Neither of the MPIs have extensive experience as clinical trial leaders. While some of the co-investigators do have relevant experience, no single individual is identified as the true lead for the clinical trial aspects of this proposal.

3. Innovation:

Strengths

- The NTrainer is unprecedented, to my knowledge, as a device for improving oromotor/feeding abilities in premature infants.

- The proposal to examine transcriptomic patterns as predictors of feeding/training readiness also is unprecedented.

Weaknesses

- A comparison of transcriptomics to clinical measures of potential feeding readiness is well within the scope of the proposal but is not put forward.

4. Approach:

Strengths

- Preliminary data suggest that NTrainer treatment is associated with better performance, both on physiological measures (non-nutritive sucking) and in clinical outcomes (full PO feeding and length of hospitalization. (Though see below for comment on sham condition.)
- Proposal to perform the study across multiple sites will support timely execution of the study and generalizability of the results.
- Feasibility is very good, as demonstrated through the preliminary results.
- Examination of multiple clinical outcomes is a strength – both oromotor function and full PO feeds

Weaknesses

- The application cites choking and aspiration as two of the primary complications of unsuccessful early feeding, but it is never argued whether improved sucking alone might affect these outcomes, which may depend as well on non-oral (laryngeal and esophageal?) functions.
- Extension to infants born at 24 and 25 weeks gestational age goes beyond the scope of the preliminary data (which starts at 26 weeks GA), and is not specifically justified in the proposal.
- For study 1, it is not clear how the presence of BPD is expected to affect the intervention. This is neither stated in the text nor accounted for in the power analyses.
- It is unclear whether the sham condition is an adequate control, both for the preliminary data and for the proposed research. Rather than just a pacifier for the sham condition, an entire sham apparatus could be considered. The caretakers/feeding personnel need to be "blinded" as much as possible.
- There is no discussion of whether the physiologic measure of NNS might be used as a predictor of oral feeding success, and how it might compare to transcriptomic data as a predictor.
- The study 3 aim of examining associations with Bayley scores represents a large leap. More proximal would be examination of weight gain and other close associates of feeding. The literature does not show that feeding issues are causative of neurodevelopmental outcomes, only that they are associated.

5. Environment:

Strengths

- Good clinical volume and trial experience at all 3 planned sites.
- Environmental support for other aspects of the trial (transcriptomics; device support, etc.) is all good.

Protections for Human Subjects:

Acceptable Risks and/or Adequate Protections

- Acceptable, but see comment on DSMP, below.

Data and Safety Monitoring Plan (Applicable for Clinical Trials Only):

Unacceptable

- The safety monitoring plan describes appropriate site-based plans, but does not explicitly describe study-wide (i.e., cross-site) monitoring. An independent data and safety monitoring committee should be instituted.

Inclusion of Women, Minorities and Children and not IRB Exemption #4.

- Sex/Gender: Distribution justified scientifically
- Race/Ethnicity: Distribution justified scientifically
- Inclusion/Exclusion of Children under 21: Including ages < 21 justified scientifically
- Appropriate

Vertebrate Animals:

- Not Applicable (No Vertebrate Animals)

Biohazards:

- Not Applicable (No Biohazards)

Budget and Period of Support:

- Recommend as Requested

Additional Comments to Applicant (Optional):

- Karen the Orangutan! Love it (Dr. Govindaswami).

CRITIQUE 3:

Significance: 2
Investigator(s): 2
Innovation: 2
Approach: 2
Environment: 2

Overall Impact: The application focuses on an extremely important problem, namely the sucking behavior of extremely preterm infants. The application lays out an ambitious plan to combine an intervention with epigenetic measures from infant saliva and long-term health and behavioral outcomes. Findings from this project will have significant and far-reaching impacts on science as well as on medical practice

1. Significance:

Strengths

- The application sets forth a bold agenda to integrate perspectives from intervention science and epigenetics, as well as from developmental science, to understand and improve the early sucking behavior of extremely premature infants and later developmental outcomes.

Weaknesses

- None noted

2. Investigator(s):

Strengths

- The main collaborators (Barlow and Maron) are highly qualified to conduct the work, as are their co-investigators

Weaknesses

- None noted

3. Innovation:

Strengths

- This is a highly innovative project. The sucking intervention (Pulsed Trainer) is ingenious and effective. The inclusion of epigenetic data from saliva as the intervention proceeds will reveal important gene by intervention interactions. The follow-up includes not only standard measures at 18 and 24 months but very important information on the experiences of the infants during the interval from NICU release.

Weaknesses

- None noted

4. Approach:

Strengths

- The methods employed are all well-described and appropriate for the questions asked.

Weaknesses

- Some more description of the SHAM condition would be helpful. Are there any implications for the nutritional intake of the two groups?

5. Environment:

Strengths

- The facilities and resources are excellent.

Weaknesses

- None noted.

Protections for Human Subjects:

- Acceptable Risks and/or Adequate Protections

Inclusion of Women, Minorities and Children and not IRB Exemption #4.

- Sex/Gender: Distribution justified scientifically
- Race/Ethnicity: Distribution justified scientifically
- Inclusion/Exclusion of Children under 21: Including ages < 21 justified scientifically
- Appropriate

Budget and Period of Support:

- Recommend as Requested

THE FOLLOWING SECTIONS WERE PREPARED BY THE SCIENTIFIC REVIEW OFFICER TO SUMMARIZE THE OUTCOME OF DISCUSSIONS OF THE REVIEW COMMITTEE, OR REVIEWERS' WRITTEN CRITIQUES, ON THE FOLLOWING ISSUES:

PROTECTION OF HUMAN SUBJECTS (Resume): ACCEPTABLE

INCLUSION OF WOMEN PLAN (Resume): ACCEPTABLE

INCLUSION OF MINORITIES PLAN (Resume): ACCEPTABLE

INCLUSION OF CHILDREN PLAN (Resume): ACCEPTABLE

COMMITTEE BUDGET RECOMMENDATIONS: The budget was recommended as requested.

Ad hoc or special section application percentiled against "Total CSR" base.

NIH has modified its policy regarding the receipt of resubmissions (amended applications). See Guide Notice NOT-OD-14-074 at http://grants.nih.gov/grants/guide/notice-files/NOT-OD-14-074.html. The impact/priority score is calculated after discussion of an application by averaging the overall scores (1-9) given by all voting reviewers on the committee and multiplying by 10. The criterion scores are submitted prior to the meeting by the individual reviewers assigned to an application, and are not discussed specifically at the review meeting or calculated into the overall impact score. Some applications also receive a percentile ranking. For details on the review process, see http://grants.nih.gov/grants/peer_review_process.htm#scoring.

MEETING ROSTER

Biobehavioral and Behavioral Sciences Subcommittee
National Institute of Child Health and Human Development Initial Review Group
EUNICE KENNEDY SHRIVER NATIONAL INSTITUTE OF CHILD HEALTH & HUMAN DEVELOPMENT
CHHD-H 1
June 10, 2015 - June 11, 2015

CHAIRPERSON

ABBEDUTO, LEONARD J., PHD
DIRECTOR, MIND INSTITUTE
PROFESSOR, PSYCHIATRY & BEHAVIORAL SCIENCES
SCHOOL OF MEDICINE
UNIVERSITY OF CALIFORNIA, DAVIS
SACRAMENTO, CA 95817

MEMBERS

BROWNELL, CELIA A, PHD
PROFESSOR
DEPARTMENT OF PSYCHOLOGY
UNIVERSITY OF PITTSBURGH
PITTSBURGH, PA 15206

CARLO, MARIA S., PHD
ASSOCIATE PROFESSOR
DEPARTMENT OF PSYCHOLOGY
UNIVERSITY OF TEXAS HEALTH SCIENCES
CENTER AT HOUSTON
HOUSTON, TX 77030

CARLSON, STEPHANIE M., PHD
PROFESSOR AND DIRECTOR OF RESEARCH
INSTITUTE OF CHILD DEVELOPMENT
UNIVERSITY OF MINNESOTA, TWIN CITIES
MINNEAPOLIS, MN 55455

COURTNEY-FARUQEE, SUSAN M, PHD *
PROFESSOR AND CHAIR
DEPARTMENT OF PSYCHOLOGICAL AND BRAIN
SCIENCES
JOHNS HOPKINS UNIVERSITY
BALTIMORE, MD 21218

DUBOW, ERIC F, PHD *
PROFESSOR
CENTER FOR FAMILY AND DEMOGRAPHIC RESEARCH
AND DEPARTMENT OF PSYCHOLOGY
BOWLING GREEN STATE UNIVERSITY
BOWLING GREEN, OH 43303

GRIGORENKO, ELENA L., PHD
EMILY FRASER BEEDE PROFESSOR
CHILD STUDY CENTER
DEPARTMENT OF PSYCHOLOGY
DEPARTMENT OF EPIDEMIOLOGY & PUBLIC HEALTH
YALE UNIVERSITY
NEW HAVEN, CT 06510

GROER, MAUREEN EDITH, PHD *
PROFESSOR
COLLEGE OF NURSING
UNIVERSITY OF SOUTH FLORIDA
TAMPA, FL 33612

HARRING, JEFFREY R., PHD
ASSOCIATE PROFESSOR
DEPARTMENT OF HUMAN DEVELOPMENT
AND QUANTITATIVE METHODOLOGY
UNIVERSITY OF MARYLAND, COLLEGE PARK
COLLEGE PARK, MD 20742

HOLDEN, GEORGE W., PHD
PROFESSOR
DEPARTMENT OF PSYCHOLOGY
DEDMAN COLLEGE
SOUTHERN METHODIST UNIVERSITY
DALLAS, TX 75275

HOLLAND, SCOTT KERRY, PHD
PROFESSOR OF RADIOLOGY,
PEDIATRICS, NEUROSCIENCE AND PHYSICS
IMAGING RESEARCH CENTER
CHILDREN'S HOSPITAL RESEARCH FOUNDATION
UNIVERSITY OF CINCINNATI
CINCINNATI, OH 45229

MENDELSOHN, ALAN L., MD
ASSOCIATE PROFESSOR OF PEDIATRICS
DEPARTMENT OF PEDIATRICS
SCHOOL OF MEDICINE AND BELLEVUE HOSPITAL
CENTER
NEW YORK UNIVERSITY
NEW YORK, NY 10016

MENON, VINOD , PHD *
PROFESSOR
DEPARTMENT OF PSYCHIATRY AND BEHAVORIAL
SCIENCE
STANFORD UNIVERSITY
STANFORD, CA 94305

MERRILL, EDWARD C, PHD *
ASSOCIATE PROFESSOR
DEPARTMENT OF PSYCHOLOGY
UNIVERSITY OF ALABAMA
TUSCALOOSA, AL 354870348

MERVIS, CAROLYN B., PHD
PROFESSOR AND DISTINGUISHED UNIVERSITY
SCHOLAR
DEPT OF PSYCHOLOGICAL & BRAIN SCIENCES
UNIVERSITY OF LOUISVILLE
LOUISVILLE, KY 40292

MORRISON, FREDERICK J., PHD
PROFESSOR
DEPARTMENT OF PSYCHOLOGY
UNIVERSITY OF MICHIGAN, ANN ARBOR
ANN ARBOR, MI 48109

PINDERHUGHES, ELLEN E., PHD
ASSOCIATE PROFESSOR
ELIOT PEARSON DEPARTMENT
OF CHILD DEVELOPMENT
TUFTS UNIVERSITY
MEDFORD, MA 02155

RUNYAN, DESMOND KIMO, MD *
EXECUTIVE DIRECTOR
DEPARTMENT OF PEDIATRICS
SCHOOL OF MEDICINE
UNIVERSITY OF COLORADO
AURORA, CO 80045

SILVERMAN, WAYNE P., PHD
DIRECTOR OF INTELLECTUAL DISABILITIES RESEARCH
PROFESSOR OF PSYCHIATRY AND BEHAVIORAL
SCIENCES
CO-DIRECTOR, INTELLECTUAL AND DEVELOPMENTAL
DISABILITIES RESEARCH CENTER
KENNEDY KRIEGER INSTITUTE
BALTIMORE, MD 21205

STEVENSON, HOWARD C. JR, PHD
CONSTANCE E. CLAYTON PROFESSOR OF URBAN
EDUCATION
APPLIED PSYCHOLOGY AND HUMAN DEVELOPMENT
DIVISION
GRADUATE SCHOOL OF EDUCATION
UNIVERSITY OF PENNSYLVANIA
PHILADELPHIA, PA 191046216

WANG, PAUL P., MD
SENIOR VICE PRESIDENT
AND HEAD OF MEDICAL RESEARCH
AUTISM SPEAKS
NEW YORK, NY 10016

SCIENTIFIC REVIEW OFFICER

HOPMANN, MARITA R., PHD
SCIENTIFIC REVIEW ADMINISTRATOR
DIVISION OF SCIENTIFIC REVIEW
NATIONAL INSTITUTE OF CHILD HEALTH
AND HUMAN DEVELOPMENT, NIH
BETHESDA, MD 20892

EXTRAMURAL SUPPORT ASSISTANT

BECKHAM, SHAYLA
EXTRAMURAL SUPPORT ASSISTANT
DIVISION OF EXTRAMURAL ACTIVITIES
NICHD/DSR
NATIONAL INSTITUTES OF HEALTH
BETHESDA, MD 20892

* Temporary Member. For grant applications, temporary members may participate in the entire meeting or may review only selected applications as needed.

Consultants are required to absent themselves from the room during the review of any application if their presence would constitute or appear to constitute a conflict of interest.
